# Diagnosis and treatment of post-traumatic stress disorder (PTSD) through the analysis of two clinical cases

**DOI:** 10.1192/j.eurpsy.2025.1964

**Published:** 2025-08-26

**Authors:** M. E. Sánchez-Escalonilla, A. Muñoz San Jose, P. Aguirre, M. Viaña, C. Herranz, I. Diéguez, Á. Esquembre, M. F. Bravo

**Affiliations:** 1 Hospital Universitario La Paz, Madrid, Spain

## Abstract

**Introduction:**

Ongoing global conflicts have significant implications for the mental health of affected populations, with PTSD being one of the most prevalent disorders among those exposed to active violence.

**Objectives:**

To understand the importance of PTSD diagnosis and treatment in individuals exposed to active violence through the review of two clinical cases.

**Methods:**

A clinical evaluation, diagnosis, treatment and follow-up of two patients (A and B) during their admission to our hospital and a literature review of diagnosis and treatment of PTSD.

**Results:**

We studied two patients from Armenia, a country currently engaged in armed conflict, who sustained injuries from a bomb explosion. Patient A had burns covering 18% of Total Body Surface Area (TBSA) and three fingers amputated, while B had burns in 50% of TBSA. Both were admitted to the Intensive Care Unit, where psychiatric evaluations were conducted, and then they were transferred to the plastic surgery unit for further care until discharge. During the first week of admission, symptoms such as flashbacks, nightmares, emotional numbness, feelings of fear, hopelessness, excessive guilt, insomnia, hypervigilance and episodes of depersonalization or derealization began to appear. Patient A exhibited an externalizing profile of symptoms (nocturnal agitation, crying and verbalization of guilt-related ruminations), while B presented an internalizing profile (affective numbness and dissociative episodes). Both cases were diagnosed with PTSD after more than a month of persistent symptoms. Psychopharmacological treatment was initiated after 20 days of hospitalization. Patient A was treated with quetiapine 200 mg/day for nocturnal agitation and sertraline 100 mg/day; while Patient B started sertraline 100mg/day and mirtazapine 15 mg/day. According to literature, first-line pharmacological treatment for PTSD includes SSRIs such as sertraline or fluoxetine. As a second-line we found mirtazapine, antipsychotics and prazosin. Benzodiazepines are not a choice and should be used cautiously (Schrader et al. MM 2021; 118 (6), 546-551). First-line treatment for PTSD is trauma-focused therapy and eye movement desensitization and reprocessing (EMDR) (Mann et al. TI 2023). In our case, such therapy was not possible to start due to language barriers and the severity of patients’ physical conditions. Notably, current clinical trials are exploring the use of psychedelics in therapy to improve PTSD symptoms (Krediet et al. IJNP 2020; 23 (6), 385-400).

**Conclusions:**

PTSD is a relatively common mental disorder, affecting up to 11% of war victims. Early detection of symptoms is crucial to start an appropiate psychotherapeutic treatment. Although psychopharmacological interventions are recommended as a second-line treatment, they may sometimes be the only feasible option, as demonstrated in these two clinical cases.

**Disclosure of Interest:**

None Declared

